# Oral Syphilis

**Published:** 1904-08

**Authors:** Albert L. Midgley

**Affiliations:** Dental Externe to Rhode Island Hospital; Assistant Dental Surgeon to St. Joseph's Hospital, Providence, R. I.


THE AMERICAN
JOURNAL OF DENTAL SCIENCE

### ORAL SYPHILIS.

ALBERT L. MIDGLEY, D. M. D.

Dental Externe to Rhode Island Hospital; Assistant Dental Surgeon to St. Joseph's Hospital, Providence, R. I.

(Written for *The American Journal of Dental Science.*)

Syphilitic lesions about the tissues of the mouth are met with very frequently in the practice of dentistry of today, and on account of the infectious nature of the disease and the grave injury to both patient and dentist through the accidental transmission of the syphilitic virus, a thorough knowledge of the pathology of syphilis is absolutely necessary. When we consider the number afflicted, and the vast amount of suffering and destruction of tissue that syphilis causes, we should be constantly on the look out for any of its lesions, and in justice to all patients, regard with suspicion any sore or ulcer found in the oral cavity.

The care of the mouths of syphilitic patients is of the utmost importance to their welfare, and with modern dental therapeutics, hygiene and prophylaxis we are in a position to aid the patient and lessen greatly the dangers of transmitting the disease.

Syphilis appears in two well marked forms, acquired and hereditary, which differ somewhat in the time, order and appearance of their lesions, yet resemble each other very closely. The course of the disease is divided into three stages, primary, secondary and tertiary, but in hereditary syphilis there is no primary stage. The first stage of the disease includes those symptoms, and pathological expressions which appear from the time of infection, up to the appearance of the symptoms of the second stage. The secondary stage begins seventy to eighty days after infection, and includes the lesions of the skin, and mucous membrane with the constitutional disturbances. Necrosis of bone, and the soft tissues is characteristic of the tertiary stage. The symptoms of this stage may appear at the latter part of the secondary or manifest themselves sometimes later.

The chancre is the only lesion of the first stage, and appears at the point of infection, after an incubation period of from 18 to 25 days. It is usually but a single lesion. We may, however, find the multiple variety which is rarely seen in the field of dental operations. An early recognition of this lesion is very essential in preventing the spread of the disease for its secretion though scanty, is exceedingly virulent.

The chancre begins as a small, round, well defined excoriated spot, with an indurated base, raised edges and depressed centre. The surface is smooth, polished and free from granulations, and its color varies from a light to a brownish red, which may turn to a coppery color, and become covered with a crust. At the same time or shortly after the appearance of the chancre, the ganglia in direct relation to the seat of infection, become enlarged. This affection of the ganglia may be only unilateral, but it is usually bilateral, with the enlargement more pronounced on the side corresponding to the seat of infection.

The following statistics may be of interest. In 9,059 cases of extra genital infection, 1,810 appeared upon the lips, 734 were found in the buccal cavity, 401 were situated upon the tongue, tonsils or gums, or in other words 32% appeared within the field of dental and oral surgery. Another fact worthy of note is that 35% of chancres of the finger, have been found among dentists.

The characteristic lesions of the second stage of the disease appearing in the mouth, are mucous patches. They are flat or slightly elevated, about ½ an inch in diameter, and vary in color from a grayish pink to a reddish brown. They begin as small red spots, oval or round in outline, and discharge a sticky foul secretion, which is very virulent and contagious. They are most frequently seen on the mucous membrane of the cheek, generally at a seat on a level with the line of occlusion of the teeth. They are also found upon the roof of the mouth, and at the angle, and also upon the tonsils, fances and sides of the tongue. The saliva itself is not infectious, but is made so by flowing over these patches, and coming in contact with the secretion. The mucous patches often increase by enormous cell development to quite a size, and then the name condylomata lata is given them.

To successfully treat these forms of syphilitic lesions, a clean condition of the mouth is essential for they flourish very well in an unclean mouth, where tartar and decaying teeth are present. With improved ideas and methods in the treatment of syphilis, lesions of the third stage of the disease are not as commonly met with now as formerly. Necrosis of bone is very characteristic of this stage of the disease, that of the hard palate especially. Syphilitic gumma of the tongue are also seen in this stage, but they are very rare.

Mercurial stomatitis is a condition not very often seen, owing to the care used at the present time in administering mercury. It is characterized by a metallic, stale odor of the breath, coated tongue and a bitter coppery taste which is more disagreeable upon awakening. The gums are puffy, have a tendency to bleed easily, and a blue line appears along the gingival border. Other symptoms are increased flow of saliva, diarrhoea, head ache and no appetite. If untreated the tongue swells; the teeth loosen and fall out; the lips and cheeks become tumid and ulcers appear in the mouth.

In the treatment of this condition, mercury should be stopped, and an astringent mouth wash prescribed. As as antisialogogue 5 minines of a solution of atropine sulfate, one grain to an ounce of water, hypodermically, every four or six hours is very good.

Hereditary syphilis closely resembles the acquired form, though there is no chancre, nor are its lesions as typical and regular in their appearance. The most important symptom is snuffles, which is due to a thickening of the nasal mucous membrane. The lips are fissured and mucous patches and condylomata are found upon the mucous membrane of the buccal cavity as in acquired syphilis.

In hereditary syphilis the teeth are important landmarks. The deciduous teeth have nothing pathognomonic about them, although in syphilitic children, they often present certain peculiarities. The superior central incisors of the permanent set are the most typical, and are of a dark color, narrow, peg shaped and broader at the cervical margin than upon the cutting edge, which is crescent in outline with the corners rounded.

The horizontal furrows which are often seen have nothing to do with syphilis, but are usually the result of some one of the diseases of childhood. In making a diagnosis, if the centrals are well marked, they should be given careful consideration, and if doubt exists, it is well to look for further evidence of syphilis.

The importance of a thorough knowledge of this disease is very evident, and if through our ability to recognize its lesions, and through our care in sterilizing instruments, we prevent the spread of the disease among our innocent patients, we are doing much to place our science on a higher and broader plane.

---

### RIGG'S DISEASE, PYORRHEA ALVEOLARIS, OR SO-CALLED INTERSTITIAL GINGIVITIS, IN A NUT SHELL.

By B. F. Arrington, D. D. S.,
Goldsboro, N. C.

(Written for *The American Journal of Dental Science.*)

